# A candidate molecular signature associated with tamoxifen failure in primary breast cancer

**DOI:** 10.1186/bcr2158

**Published:** 2008-10-17

**Authors:** Julie A Vendrell, Katherine E Robertson, Patrice Ravel, Susan E Bray, Agathe Bajard, Colin A Purdie, Catherine Nguyen, Sirwan M Hadad, Ivan Bieche, Sylvie Chabaud, Thomas Bachelot, Alastair M Thompson, Pascale A Cohen

**Affiliations:** 1 Université de Lyon, 69008 Lyon, France; 2 Université de Lyon, Lyon 1, ISPB, Faculté de Pharmacie de Lyon, 69008 Lyon, France; 3 INSERM, U590, 69008 Lyon, France; 4 Centre Léon Bérard, FNCLCC, 69373 Lyon, France; 5 Department of Surgery and Molecular Oncology, Ninewells Hospital and Medical School, University of Dundee, Dundee DD1 9SY, UK; 6 CNRS UMR 5160, Centre de Pharmacologie et Biotechnologie pour la Santé, Faculté de Pharmacie, 34090 Montpellier, France; 7 Division of Pathology and Neuroscience, Ninewells Hospital and Medical School, University of Dundee, Dundee DD1 9SY, UK; 8 Centre de Biochimie Structurale, CNRS, INSERM, Université Montpellier I, 34090 Montpellier, France; 9 Centre Léon Bérard, FNCLCC, Unité de Biostatistique et d'Evaluation des Thérapeutiques, 69373 Lyon, France; 10 INSERM ERM206, Laboratoire TAGC, Université d'Aix-Marseille II, 13288 Marseille Cedex 9, France; 11 INSERM U735, Centre René Huguenin, FNCLCC, 92210 St-Cloud, France; 12 Centre Léon Bérard, FNCLCC, Département de Médecine, 69373 Lyon, France

## Abstract

**Introduction:**

Few markers are available that can predict response to tamoxifen treatment in estrogen receptor (ER)-positive breast cancers. Identification of such markers would be clinically useful. We attempted to identify molecular markers associated with tamoxifen failure in breast cancer.

**Methods:**

Eighteen initially ER-positive patients treated with tamoxifen requiring salvage surgery (tamoxifen failure [TF] patients) were compared with 17 patients who were disease free 5 years after surgery plus tamoxifen adjuvant therapy (control patients). cDNA microarray, real-time quantitative PCR, and immunohistochemistry on tissue microarrays were used to generate and confirm a gene signature associated with tamoxifen failure. An independent series of 33 breast tumor samples from patients who relapsed (*n* = 14) or did not relapse (*n* = 19) under tamoxifen treatment from a different geographic location was subsequently used to explore the gene expression signature identified.

**Results:**

Using a screening set of 18 tumor samples (from eight control patients and 10 TF patients), a 47-gene signature discriminating between TF and control samples was identified using cDNA arrays. In addition to *ESR1*/*ERα*, the top-ranked genes selected by statistical cross-analyses were *MET*, *FOS*, *SNCG*, *IGFBP4*, and *BCL2*, which were subsequently validated in a larger set of tumor samples (from 17 control patients and 18 TF patients). Confirmation at the protein level by tissue microarray immunohistochemistry was observed for ER-α, γ-synuclein, and insulin-like growth factor binding protein 4 proteins in the 35 original samples. In an independent series of breast tumor samples (19 nonrelapsing and 14 relapsing), reduced expression of *ESR1*/*ERα*, *IGFBP4*, *SNCG*, *BCL2*, and *FOS *was observed in the relapsing group and was associated with a shorter overall survival. Low mRNA expression levels of *ESR1*/*ERα*, *BCL2*, and *FOS *were also associated with a shorter relapse-free survival (RFS). Using a Cox multivariate regression analysis, we identified *BCL2 *and *FOS *as independent prognostic markers associated with RFS. Finally, the *BCL2*/*FOS *signature was demonstrated to have more accurate prognostic value for RFS than *ESR1*/*ERα *alone (likelihood ratio test).

**Conclusions:**

We identified molecular markers including a *BCL2*/*FOS *signature associated with tamoxifen failure; these markers may have clinical potential in the management of ER-positive breast cancer.

## Introduction

Breast cancer remains a global public health problem, with some 1.1 million women newly diagnosed with breast cancer in 2002 [1]. Nevertheless, there has been a decline in breast cancer mortality in the Western world over the past decade, which is at least in part attributable to the use of tamoxifen adjuvant therapy [2,3]. For estrogen receptor (ER)-positive cancers, 5 years of adjuvant tamoxifen therapy reduces the annual breast cancer death rate by 31%, with a persistent cumulative effect even 15 years after primary treatment [3]. Impressive early data with tamoxifen in the adjuvant setting led clinicians to use tamoxifen as neoadjuvant therapy to avoid surgery in elderly women with ER-positive cancer [4]. However, long-term follow up and clinical trials demonstrated that up to 62% of cancers initially responsive to endocrine therapy subsequently escaped control, with the patient then requiring salvage surgery [4,5]. Thus, the use of tamoxifen as primary endocrine therapy has been reserved for patients who decline or are unfit for surgery as first-line therapy.

Although aromatase inhibitors may replace tamoxifen as first-line neoadjuvant and adjuvant endocrine therapy for most postmenopausal women, tamoxifen will continue to play a role in premenopausal women as a second-line therapy in postmenopausal women and in chemoprevention for all age groups [6]. However, the molecular mechanisms that are involved in the chemoresistance to tamoxifen remain unclear; understanding such processes could benefit clinical decision making.

Recent advances in genomics have provided tools that allow gene expression profiling of solid tumors. Various studies examining gene expression profiles of breast cancer have allowed the molecular classification of clinically distinct subclasses of tumors [7,8] and the identification of molecular markers associated with prognosis/clinical outcome [9-11] and of predictive signatures that relate to therapeutic response [12,13]. In this study our aims was to identify a set of candidate molecular markers associated with failure of tamoxifen treatment and that can discriminate between patients with tamoxifen-sensitive breast cancer and those with tamoxifen-resistant breast cancer. These molecular markers could be useful in a clinical setting to plan patient management based on tumor biology. To achieve our objectives, we used a variety of techniques: cDNA arrays to identify a discriminatory gene expression signature, real-time quantitative PCR (RTQ-PCR) to examine gene expression at the transcript level, and tissue microarrays (TMAs) with immunohistochemistry to look at protein expression levels of the candidate markers in a first cohort of breast tumor samples. An independent cohort of patients from a different geographical location was then used to assess, using RTQ-PCR, the pertinence of the molecular markers identified. This work presents a step toward using molecular markers of tamoxifen failure as tools of clinical utility.

## Materials and methods

### Cohort of patients and breast tumor samples used for biomarker identification

A first cohort of 35 postmenopausal Caucasian women (age range 41 to 92 years; median age 74 years) with ER-α-positive primary breast cancer diagnosed at the Ninewells Hospital (Dundee, UK), for whom complete clinical and pathological data were available, were studied (Table 1). Eighteen women were considered initially unfit for surgery or declined surgical intervention and received neoadjuvant tamoxifen therapy only (20 mg/day). Tumors were monitored by clinical caliper measurement and mammography. After an initial response, as indicated by a clinical and mammographic reduction in tumor size, the tumors increased in size despite tamoxifen therapy and were removed by surgical excision (tamoxifen failure [TF] group; *n* = 18) and used in the study. No tissue from these patients prior to endocrine therapy was available. Breast tumor samples from the control group (age-matched women selected from the same geographic and ethnic population who underwent surgical resection before any endocrine therapy, and who did not exhibit any cancer recurrence for 5 years after primary surgical resection and subsequent adjuvant treatment with tamoxifen alone) were excised. Using the χ^2 ^test, there was no significant difference in age, histologic grade, lymph node status, or tumor size between the two groups (Table 1). Informed consent was obtained from all patients, and the study was approved by the ethics committee of the institution.

**Table 1:** Initial cohort of patients (Ninewells Hospital, Dundee, UK): characteristics of the 35 patients with initially ER-positive primary breast cancer

Characteristic	Control group (*n* = 17)	TF group (*n* = 18)	*P*^a^
Age (years)			
<70	5	4	NS (0.92)
≥ 70	12	14	
Histological grade^b^			
I + II	14	8	NS (0.11)
III	3	8	
Lymph node status			
Node negative	10	4	NS (0.06)
Node positive	7	14	
Macroscopic tumor size^c^			
<30 mm	11	6	NS (0.17)
≥ 30 mm	6	11	

^a^*P*-value (χ^2 ^test) was considered significant when *P *< 0.05. ^b^Information available for 33 patients. ^c^Information available for 34 patients. ER, estrogen receptor; NS, not significant; TF, tamoxifen failure.

From this cohort, 18 tumor samples (from eight control patients and 10 TF patients), for which sufficient total RNA could be obtained, were used in the initial cDNA array investigation; the whole set (17 control samples and 18 TF samples) was used for RTQ-PCR measurements; and 33 samples were used to conduct the TMA experiments (16 control samples and 17 TF samples; formalin-fixed paraffin-embedded blocks were not available for two patients).

### Total RNA extraction

Surgical resection specimens were transported fresh to the adjacent pathology laboratory, and small pieces of tumor tissue were dissected out by a pathologist and snap frozen in liquid nitrogen before storage at -80°C. Approximately 10 mg tissue was homogenized in 750 μl QIAzol lysis reagent (Qiagen Ltd, Crawley, West Sussex, UK). RNA quality was assured using the BioAnalyzer 2100™ (Agilent Technologies, Palo Alto, CA, USA).

### Transcriptome study by cDNA-array technology

Eighteen tumor samples (from eight control patients and 10 TF patients) were used in cDNA array analysis (screening set). Gene expression levels were determined using large-scale measurement experiments using customized nylon cDNA arrays (7.5 × 11.5 cm; 1,034 human genes; 12 genes/cm^2^) produced in our facility (TAGC Laboratory, University of Aix-Marseille II, France), as previously described [14-16]. Following hybridization to each array with a ^33^P-labeled probe synthesized by reverse transcribing 5 μg total RNA [14], hybridization signals were scanned with a FUJI BAS 5000 beta imager (Raytest, Asnieres, France) and then quantified with the BZScan software, in accordance with the developers' recommendations [17] (TAGC Laboratory, Marseille, France). Intensity values were then adjusted using a normalization step based on the DNA quantification of each spot and the sum of intensities detected in each experiment.

We conducted supervised analyses of genes that could allow discrimination between the two classes of tumor samples (control and TF) by cross-analyzing the results given by three independent methods: supervised analysis using a signal-to-noise metric [18]; significance analysis of microarrays [19]; and Mann-Whitney test (*P *< 0.05). For each method, we considered the ranks assigned for each gene, and we selected the genes with the best sum of the ranks obtained using the three methods. Expression profiles were then analyzed by hierarchical clustering using the Cluster program developed by Eisen and colleagues [20], and the results visualized using Treeview software (Eisen Laboratory, Berkeley, CA, USA).

### Real-time quantitative PCR analysis

Using RTQ-PCR, tumor samples were examined as previously described [15], using a LightCycler^® ^1.5 (Roche, Meylan, France) in combination with the LightCycler Faststart DNA Master Sybr Green I (Roche), in accordance to the manufacturer's recommendations. For each gene, the amount of target was calculated as follows by normalization to the expression of the *28S *gene and relative to the calibrator: E^-(ΔCTsample-ΔCTcalibrator)^, where E is the efficiency of the RTQ-PCR reaction calculated with the slope of the corresponding standard curve, C_T _is the threshold cycle, and ΔC_T _is (C_T _target gene – C_T _28S). Statistical analysis of RTQ-PCR measurements was performed using the Mann-Whitney test and the Spearman's rank test, by Statgraphics^® ^3 plus software (Statgraphics Centurion, Herndon, VA, USA). The results were judged to be statistically significant at a confidence level greater than 95% (*P <*0.05).

### Tissue microarray experiments

Thirty-three tumor samples (16 control and 17 TF) were used to construct a TMA, containing up to six 0.6 mm diameter cores from each invasive breast tumor using a manual tissue arrayer (Beecher Instruments Inc., Sun Prairie, WI, USA). Briefly, hemotoxylin and eosin stained tumor sections were reviewed by a single pathologist (KER), and areas suitable for inclusion in the TMA marked. Sections were matched to their corresponding wax blocks (the donor blocks), and 0.6 mm diameter cores of tumor were removed from these donor blocks and inserted into the recipient paraffin TMA block in a grid arrangement.

Four micrometer sections were cut from the TMA block and placed onto poly-L-lysine coated glass slides (VWR International Ltd., Lutterworth, UK) and dried for 1 hour at 60°C, before being de-paraffinized in Histoclear (National Diagnostics, Hessle, UK) and rehydrated through a graded alcohol series. Citric acid buffer (10 mmol/l, pH 6.0) was used as a standard microwave-based antigen retrieval method. Sections were microwaved in a microwave compatible pressure vessel for 15 minutes before being immunostained on a Dako Autostainer Universal Staining System (Dako, Ely, UK) using Vectastain^® ^ABC kits (Vector Labs, Peterborough, UK), in accordance with the manufacturer's protocol. Briefly, sections were blocked by either normal goat or horse serum containing 10% (vol/vol) from stock avidin solution (Vector Labs) for 20 minutes followed by incubation with primary antibody, including 10% (vol/vol) from stock biotin solution (Vector Labs) for 1 hour to reduce nonspecific background staining. The following anti-human antibodies were used as primary antibodies: anti-insulin-like growth factor binding protein 4 (anti-IGFBP4; ab4252); anti-c-Fos (ab7963); anti-γ-synuclein (anti-SNCG; ab6169; Abcam Ltd, Cambridge, UK); anti-Bcl2 (clone 124; Dako); anti-ER-α clone 6F11 (Vision BioSystems, Newcastle-upon-Tyne, UK); and anti-c-Met (CVD13; Zymed^® ^Laboratories Inc., Paisley, UK). Sections were then incubated with either biotinylated anti-rabbit or anti-mouse antibody for 30 minutes followed by Vectastain^® ^Elite ABC reagent for another 30 minutes. Liquid diaminobenzidine (Dako) was used as a chromogenic agent for 5 minutes and sections were counterstained with Mayer's hematoxylin. In between each immunostaining step, slides were washed briefly in Tris-buffered saline (pH 7.6). Sections known to stain positively were included in each batch, and negative controls were prepared by replacing the primary antibody with Tris-buffered saline.

TMA scoring was carried out independently by one of the authors (KER, SMH), and concordance was confirmed by a specialist breast pathologist (CAP) using a Nikon Eclipse E600 light microscope. Antibody staining of cores containing tumor were assessed using a scoring system based on the quickscore method [21]. Briefly, the proportion of positive cells was estimated and given a score on a scale from 1 to 6 (1 = 0% to 4%; 2 = 5% to 19%; 3 = 20% to 39%; 4 = 40% to 59%; 5 = 60% to 79%; and 6 = 80% to 100%). The average intensity of the positively staining cells was estimated and given a score from 0 to 3 (0 = no staining; 1 = weak staining; 2 = intermediate staining; and 3 = strong staining). A quickscore was then calculated by multiplying the percentage of cells staining score by the intensity score, to yield a minimum value of 0 and a maximum value of 18.

### Independent cohort of breast tumor samples of tamoxifen failure

A separate cohort of 33 Caucasian women (age range 31 to 77 years; median age 55.5 years) with ER-positive primary breast cancer diagnosed at Centre Léon Bérard (Lyon, France) were selected (Table 2) and provided by the Centre de Ressources Biologiques of the Centre Léon Bérard (Lyon, France). The breast tumor samples were excised from women who did not receive endocrine therapy, chemotherapy, or radiotherapy before surgery. Complete clinical, histologic, and biologic information was available. All patients received postoperative adjuvant endocrine therapy alone for 5 years (tamoxifen 20 mg/day) and no chemotherapy. Fourteen patients relapsed under tamoxifen treatment (relapsing group) and 19 patients did not have a recurrence after 5 years of tamoxifen treatment (nonrelapsing group). Informed consent was obtained from all patients. Using the χ^2 ^test, there were no significant differences between groups in age, histologic grade, lymph node status, or tumor size (Table 2).

**Table 2:** Independent cohort of patients used for validation (Centre Léon Bérard, Lyon, France): characteristics of the 33 patients with initially ER-positive primary breast cancer

Characteristic	Control group (*n* = 19)	TF group (*n* = 14)	*P*^a^
Age (years)			
<70	16	8	NS (0.18)
≥ 70	3	6	
Histological grade			
I + II	12	8	NS (1)
III	7	6	
Lymph node status			
Node negative	0	0	NS (1)
Node positive	19	14	
Macroscopic tumor size			
<30 mm	10	9	NS (0.75)
≥ 30 mm	9	5	

^a^*P *value (χ^2 ^test) was considered significant when *P *< 0.05. ER, estrogen receptor; NS, not significant; TF, tamoxifen failure.

RNA extraction was performed as described above. RTQ-PCR experiments were conducted using a LightCycler 480^® ^(Roche) in combination with the LC480 SybrGreen I Master Mix (Roche), in accordance with the manufacturer's recommendations. The expression of the six genes was investigated using the same pair of primers, and the same calculation and normalization methods as described above. Statistical analysis of RTQ-PCR measurements was performed using the Mann-Whitney test using the Statgraphics^® ^3 plus software (Statgraphics Centurion). The results were judged statistically significant at a confidence level greater than 95% (*P <*0.05).

For each gene, the 33 ER-positive breast tumors were then divided into two groups: one of 16 tumors with 'low' mRNA level (lower than the median of the mRNA levels of the 33 breast tumor samples) and another of 17 tumors with 'high' mRNA level (higher than the median of the mRNA levels of the 33 breast tumor samples). Outcomes of interest were overall survival (OS) and relapse-free survival (RFS). OS was measured from the date of diagnosis to death or censored at the last follow up. RFS was measured from the date of diagnosis to relapse or censored at the last follow-up. Survival distributions were estimated using the Kaplan-Meier method and the significance of differences between survival rates was ascertained by the log-rank test, using the SPSS^® ^Software (SPSS Inc., Chicago, IL, USA). Candidate prognostic factors for RFS with a 0.05 significance level in univariate analysis were entered in a multivariate Cox model, and a backward selection procedure was used to build the final model [22]. Likelihood ratio test was used to select the best fit between models [23].

### General considerations in statistical analyses

Because this study is an exploratory analysis, all of the statistical analyses performed in this work were done at the 0.05 significance level, and no correction was applied for multiple testing.

## Results

### Identification of a discriminating 47-gene signature associated with tamoxifen failure

**Figure 1:**
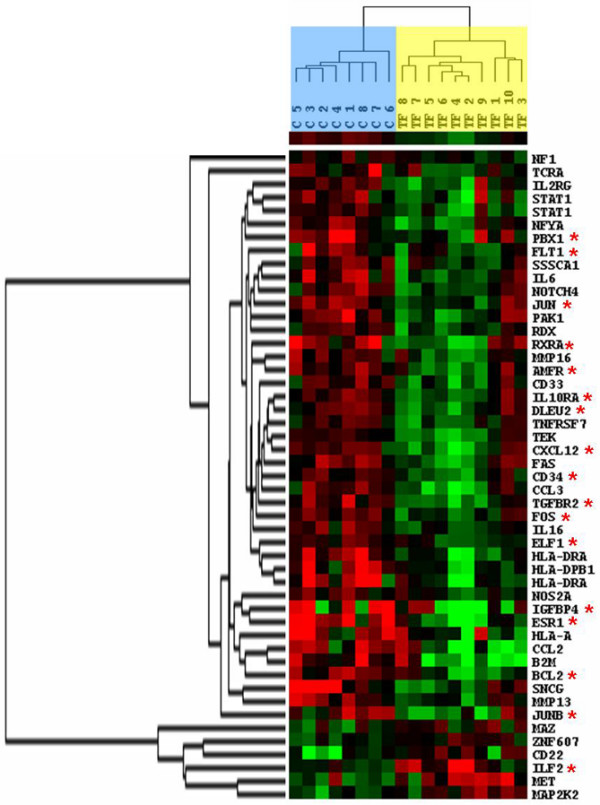
Dendogram of 18 tumor samples (eight control and 10 TF). Using the Cluster and Treeview software packages, the samples were ordered according to their degree of similarity after hierarchical clustering of the expression profiles of the 49 selected genes. Each column represents a tumor sample and each row a single gene. Expression levels above the median are presented in red and expression levels below the median are presented in green. The control tumor samples are colored blue, and the TF tumor samples yellow. Red asterisks represented genes whose expression is regulated by estradiol treatment in breast cancer cell lines [15,24-31]. C, control; TF, tamoxifen failure.

cDNA arrays were used to identify candidate genes associated with tamoxifen failure. From a training set of 18 tumor samples (eight control and 10 TF), total RNA was extracted from each sample and used to synthesize the corresponding complex probe to be hybridized on the cDNA arrays. With the aim being to identify a molecular signature that might allow discrimination between the two classes of tumor samples (control and TF), a cross-analysis based on three different statistical methods (significance analysis of microarrays, signal-to-noise statistic method, and Mann-Whitney test) was applied to the normalized cDNA array data. For each method, the ranks assigned to each gene were considered, and the genes selected with the best sum of the ranks obtained using the three methods. Forty-nine discriminant genes arose, and hierarchical clustering of the gene expression profiles discriminated between the two groups of patients (control and TF; Figure 1). Two out of the 49 discriminating genes (*HLA-DRA *and *STAT1*) were selected by two individual spots located at different places on the array, emphasizing the reproducibility of the gene signature identified.

Functional annotation of the 47 genes in the signature showed that 34% of the genes were involved in immune response (*B2M*, *CCL2*, *CCL3*, *CD22*, *CD33*, *CXCL12*, *HLA-A*, *HLA-DPB1*, *HLA-DRA*, *IL10RA*, *IL16*, *IL2RG*, *IL6*, *ILF2*, *TCRA*, and *TNFRSF7*), 23% in transcription regulation (*ELF1*, *ESR1*/*ERα*, *FOS*, *JUN*, *JUNB*, *MAZ*, *NFYA*, *PBX1*, *RXRA*, *STAT1*, and *ZNF607*), 23% in cellular proliferation regulation and mitosis control (*DLEU2*, *IGFBP4*, *MAP2K2*, *MET*, *NF1*, *NOTCH4*, *PAK1*, *SNCG*, *SSSCA1*, *TEK*, and *TGFBR2*), 9% in tumor invasion (*AMFR*, *FLT1*, *MMP13*, and *MMP16*), 4% in apoptosis (*BCL2 *and *FAS*), 4% in cell adhesion (*CD34 *and *RDX*), and 3% in other functions. Sixteen genes encoded proteins of the immune system: major histocompatibility complex proteins (*B2M*, *HLA-A*, *HLA-DPB1*, *HLA-DRA*, and *TCRA*), cell antigen (*CD22 *and *CD33*), receptors (*IL2RG*, *IL10RA*, and *TNFRSF7*), cytokines and chemokines (*IL6*, *IL16*, *CCL2*, *CCL3*, and *CXCL12*), and a transcription factor (*ILF2*). In addition, three of the 23% of genes involved in transcription regulation were genes that take part in the activator protein-1 transcription complex (*FOS*, *JUN*, and *JUNB*). Interestingly, expression of 17 genes from the 47-gene signature selected in our study (Figure 1 [red asterisks]) was associated with estrogen action, because their expression was modulated by estradiol treatment in breast cancer cells *in vitro *[15,24-31]. This suggests that identification of estradiol-regulated genes [32] or tamoxifen-regulated genes [33]*in vitro *might be a good approach to selecting prognostic or predictive molecular markers of ER-positive breast cancer [32-34].

### Real-time quantitative PCR investigation of the six top-ranked genes: *ESR1/ERα*, *MET*, *FOS*, *SNCG*, *IGFBP4*, and *BCL2*

Considering the rank assigned to each gene following the statistical cross-analysis performed on the cDNA array data (as described in the Materials and methods section [above]), *ESR1*/*ERα *emerged as the most discriminating gene, as expected [35-38]. We then investigated the expression of *ESR1*/*ERα *and that of the five following top-ranked genes (*MET*, *FOS*, *SNCG*, *IGFBP4*, and *BCL2*) by RTQ-PCR on the initial set of 18 tumor samples (eight control and 10 TF). Using Spearman rank correlation, for five genes (*ESR1*/*ERα*, *FOS*, *IGFBP4*, *MET*, and *SNCG*) the data demonstrated a positive and significant (*P *< 0.05) correlation between mRNA levels measured by cDNA array and RTQ-PCR, indicating consistency between the cDNA array and the RTQ-PCR measurements (Table 3).

**Table 3:** Spearman rank correlation between the cDNA-array and the RTQ-PCR measurements

	Spearman correlation	
	
Gene	*r*^a^	*P*^b^
*ESR1*	0.93	0.0003
*FOS*	0.89	0.0006
*IGFBP4*	0.86	0.0008
*MET*	0.82	0.003
*SNCG*	0.78	0.005
*BCL2*	0.38	NS (0.14)

^a^Spearman rank correlation coefficient. ^b^*P *value (Spearman rank correlation test) was considered significant when *P *< 0.05. NS, not significant.

To explore the reliability of the expression signature previously identified by cDNA array, RTQ-PCR of *ESR1*/*ERα*, *FOS*, *IGFBP4*, *MET*, *BCL2*, and *SNCG *gene expression was examined in a larger set of 35 tumor samples (17 control and 18 TF), including the 18 samples used in the initial screen. The data presented in Table 4 demonstrate a significant difference of expression between the control and TF groups for the six genes (Mann-Whitney test). As expected, *ESR1*/*ERα *emerged as the most significant gene (*P *< 10^-6^). mRNA levels of four genes (*IGFBP4*, *SNCG*, *BCL2*, and *FOS*) were significantly lower in the TF groups, whereas *MET *mRNA levels were significantly higher in the TF group.

**Table 4:** Statistical comparison of the mRNA levels measured by RTQ-PCR between the control and TF groups of tumor samples (Ninewells Hospital, Dundee, UK)

Genes	Tumor samples	Number of samples	mRNA levels (arbitrary units)		
			
			Median	Range	*P*^a^
*ESR1*	Control	17	72.71	28.14 to 261.30	<10^-6^
	TF	18	1.98	0.13 to 20.85	
*IGFBP4*	Control	17	2.53	1.44 to 5.93	0.0004
	TF	18	1.12	0.15 to 3.24	
*MET*	Control	17	0.48	0.15 to 2.46	0.009
	TF	18	1.23	0.18 to 12.51	
*FOS*	Control	17	0.36	0.04 to 2.54	0.02
	TF	18	0.15	0.02 to 1.46	
*SNCG*	Control	17	0.78	0.08 to 34.01	0.036
	TF	18	0.31	0.05 to 4.39	
*BCL2*	Control	17	2.87	0.08 to 7.14	0.04
	TF	18	1.08	0.13 to 6.8	

^a^*P *value (Mann-Whitney test) was considered significant when *P *< 0.05. TF, tamoxifen failure.

Using the Mann-Whitney test, the expression of the six genes was compared with clinical and pathological parameters Table 1). *ESR1*/*ERα *(*P *= 0.002) and *BCL2 *(*P *= 0.003) genes had significantly higher expression in axillary node metastasis-negative patients (*n* = 14) than in node-positive patients (*n* = 21). Moreover, gene expression levels of *ESR1*/*ERα *(*P *= 0.004), *BCL2 *(*P *= 0.003), and *FOS *(*P *= 0.01) were significantly lower in grade III (*n* = 11) than in grade I + II (*n* = 22) tumor samples. Finally, *MET *mRNA levels were significantly increased (*P *= 0.016) in tumor grade III samples compared with grade I + II samples.

### Immunohistochemical examination of ER-α, c-Fos, IGFBP4, c-Met, SNCG, and Bcl2 on tissue microarray

**Figure 2:**
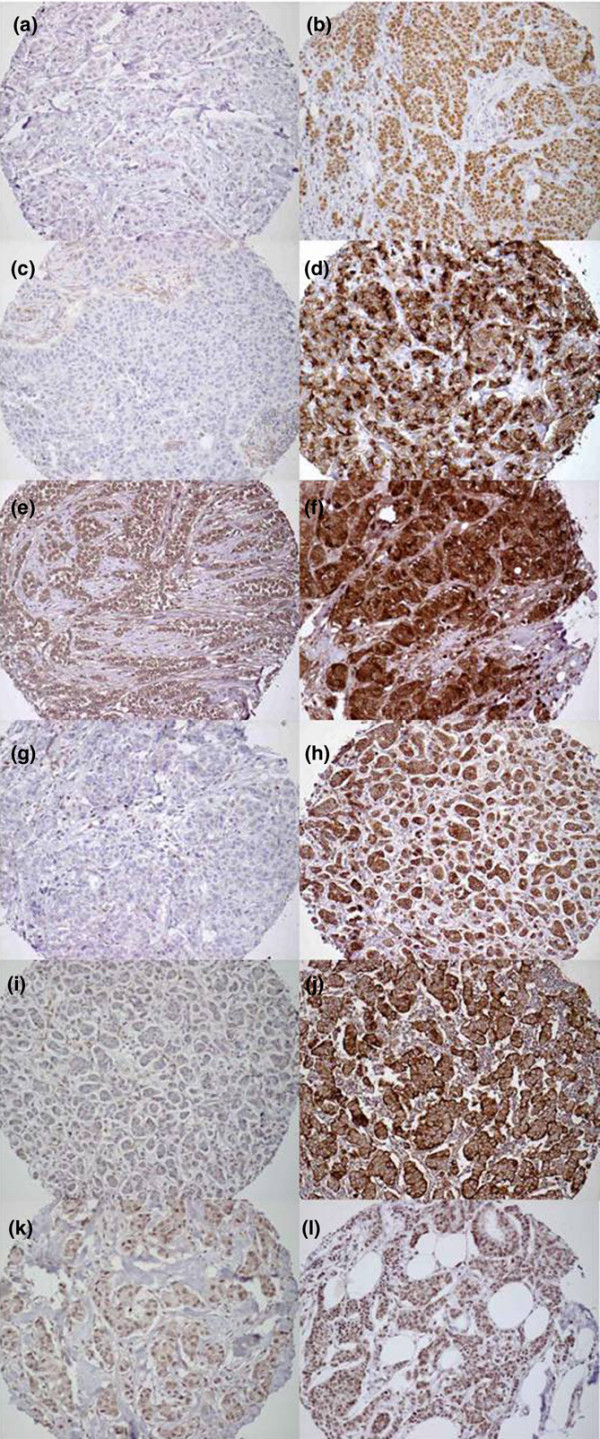
Example cores of breast tumors from the TMA. Presented are example cores of breast tumors from the TMA showing immunohistochemical staining for **(a, b) **ER-α, **(c, d) **IGFBP4, **(e, f) **SNCG, **(g, h) **Bcl2, **(i, j) **c-Met, and **(k, l) **c-Fos. Panel a shows weak staining for ER-α, b strong positive staining for ER-α, c negative staining for IGFBP4, d strong staining for IGFBP4, e weak positive staining for SNCG, f strong positive staining for SNCG, g negative staining for Bcl2, h strong positive staining for Bcl2, i negative staining for c-Met, j strong positive staining for c-Met, k negative staining for c-Fos, and l strong positive staining for c-Fos. All images were taken at ×40 objective. ER, estrogen receptor; IGFBP, insulin-like growth factor binding protein; SNCG, γ-synuclein; TMA, tissue microarray.

In order to explore, at the protein level, the expression variations of the six top-ranked genes and to ascertain whether immunohistochemical detection of these proteins on tissue sections could differentiate between the control and the TF groups, immunohistochemistry was performed on TMAs containing cores of tissue from 33 out of the 35 patients (16 control and 17 TF; Figure 2). For two samples (one control and one TF), tumor sections were not available. A Spearman rank correlation test between the TMA data and the RTQ-PCR measurements revealed a significant and positive correlation for ESR1/ER-α (*P *< 10^-4^), Bcl2 (*P *= 0.003), and IGFBP4 (*P *= 0.05; data not shown). The association of protein immunohistochemical detection with the control or TF group was studied using the Fisher exact test (Table 5). There was a significant statistical association between ER-α (*P *= 0.0004), SNCG (*P *= 0.02), and IGFBP4 (*P *= 0.03) immunohistochemical staining with patient group (control or TF). No significant association was observed for c-Fos, c-Met, and Bcl2 protein detection (*P *> 0.05).

**Table 5:** Statistical comparison of protein expression measured by TMA between the control and TF groups of tumor samples (*n *= 33; Ninewells Hospital, Dundee, UK)

Proteins	Quickscore	Samples (*n* [%])		*P*^a^
			
		Control	TF	
ER-α	0 and <4	1 (6)	11 (65)	0.0004
	From 4 to <8	4 (25)	4 (24)	
	From 8 to <12	5 (31)	2 (12)	
	From 12 to 18	6 (38)	0 (0)	
SNCG	0 and <4	0 (0)	0 (0)	0.02
	From 4 to <8	6 (38)	7 (41)	
	From 8 to <12	1 (6)	7 (41)	
	From 12 to 18	9 (56)	3 (18)	
IGFBP4	0 and <4	5 (31)	8 (47)	0.03
	From 4 to <8	4 (25)	5 (29)	
	From 8 to <12	1 (6)	4 (24)	
	From 12 to 18	6 (38)	0 (0)	
c-Fos	0 and <4	1 (6)	0 (0)	NS (0.08)
	From 4 to <8	7 (44)	2 (12)	
	From 8 to <12	1 (6)	2 (12)	
	From 12 to 18	7 (44)	13 (76)	
c-Met	0 and <4	1 (6)	0 (0)	NS (0.29)
	From 4 to <8	1 (6)	4 (24)	
	From 8 to <12	6 (38)	8 (47)	
	From 12 to 18	8 (50)	5 (29)	
Bcl2	0 and <4	5 (31)	7 (41)	NS (0.78)
	From 4 to <8	4 (25)	2 (12)	
	From 8 to <12	1 (6)	2 (12)	
	From 12 to 18	6 (38)	6 (35)	

^a^*P *value (Fisher exact test) was considered significant when *P *< 0.05. ER, estrogen receptor; IGFBP, insulin-like growth factor binding protein; NS, not significant; SNCG, γ-synuclein; TF, tamoxifen failure; TMA, tissue microarray.

### Real-time quantitative PCR investigation of *ESR1/ERα*, *MET*, *FOS*, *SNCG*, *IGFBP4*, and *BCL2 *in an independent cohort of breast tumor samples

We examined an independent cohort of ER-positive breast cancer patients from a different geographic location (Centre Léon Bérard, Lyon, France) to assess the pertinence of the biomarkers identified in the study. The separate cohort (*n* = 33) included 14 patients who relapsed under tamoxifen treatment (relapsing group) and 19 patients who did not relapse after 5 years of tamoxifen treatment (nonrelapsing group). In this cohort, low mRNA levels of *FOS*, *BCL2*, *SNCG*, and *IGFBP4 *were significantly associated with tamoxifen failure (*P *< 0.05, Mann-Whitney test; Table 6). *MET *was the only biomarker that could not be confirmed.

**Table 6:** Statistical comparison of the mRNA levels measured by RTQ-PCR between the nonrelapsing and relapsing groups of tumor samples from the independent cohort (Centre Léon Bérard, Lyon, France)

Genes	Tumor samples	Number of samples	mRNA levels (arbitrary units)		
			
			Median	Range	*P*^a^
*ESR1*	Nonrelapsing	19	117.9	0.33 to 472.35	0.0001
	Relapsing	14	2.55	0.03 to 51.75	
*IGFBP4*	Nonrelapsing	19	97.52	0.02 to 12691.41	0.02
	Relapsing	14	1.40	0.01 to 7282.33	
*MET*	Nonrelapsing	19	0.10	0.08 to 10.26	NS (0.33)
	Relapsing	14	0.12	0.10 to 2.28	
*FOS*	Nonrelapsing	19	8.72	0.03 to 47.92	0.0001
	Relapsing	14	0.28	0.01 to 1.46	
*SNCG*	Nonrelapsing	19	5.99	0.04 to 915.74	0.005
	Relapsing	14	0.52	0.01 to 18.28	
*BCL2*	Nonrelapsing	19	5.59	0.01 to 17.24	0.0004
	Relapsing	14	0.12	0.01 to 8.57	

^a^*P *value (Mann-Whitney test) was considered significant when *P *< 0.05. NS, not significant; RTQ-PCR, real-time quantitative PCR.

### Prognosis significance of the biomarkers identified

**Figure 3:**
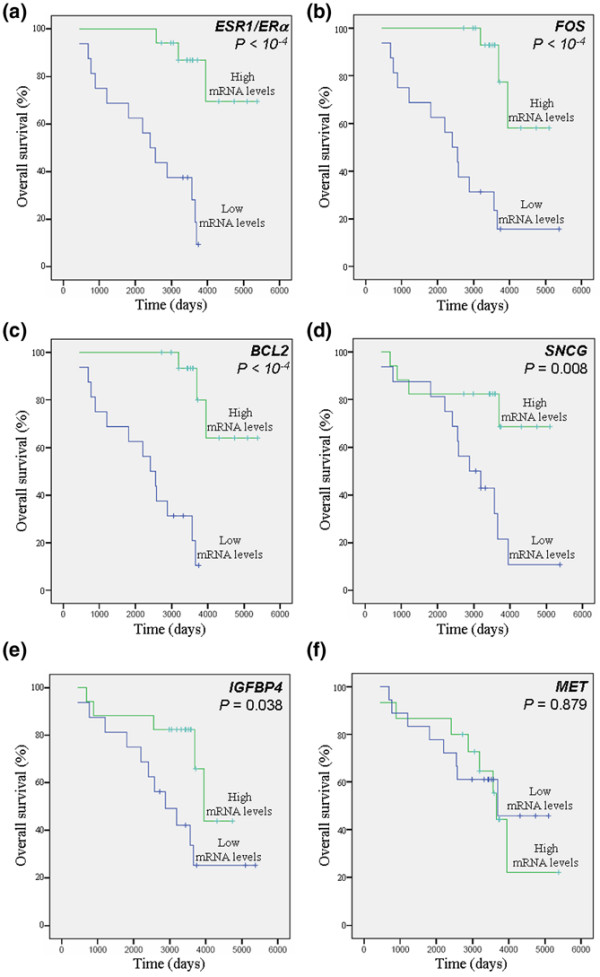
Overall survival. Presented are Kaplan-Meier curves (log-rank test analysis) of breast cancer samples from the Centre Léon Bérard cohort. Effect of **(a) ***ESR1*/*ERα*, **(b) ***FOS*, **(c) ***BCL2*, **(d) ***SNCG*, **(e) ***IGFBP4*, and **(f) ***MET *mRNA levels on overall survival among the 33 breast cancer samples.

**Figure 4:**
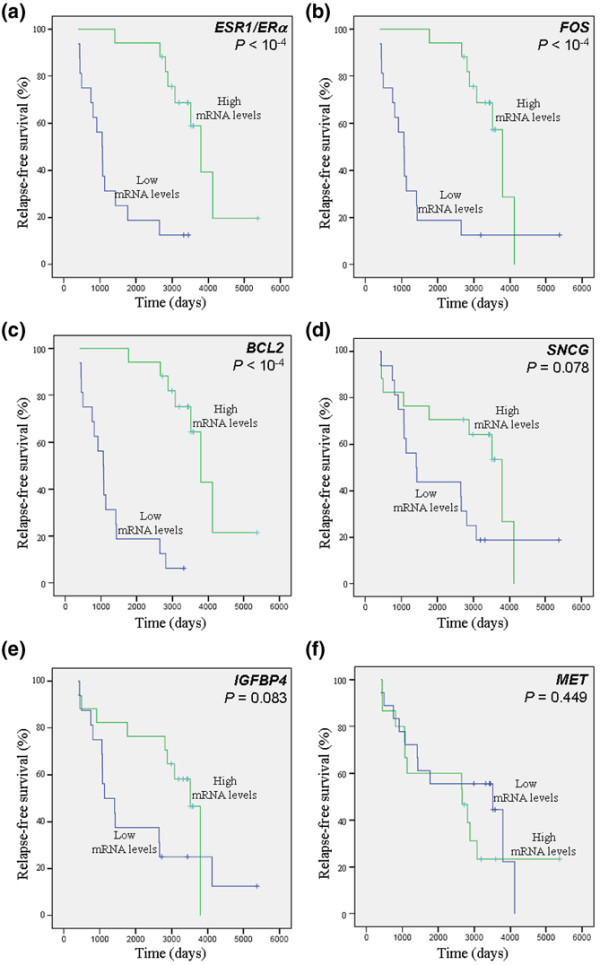
Relapse-free survival. Presented are Kaplan-Meier curves (log-rank test analysis) of breast cancer samples from the Centre Léon Bérard cohort. Effect of **(a) ***ESR1*/*ERα*, **(b) ***FOS*, **(c) ***BCL2*, **(d) ***SNCG*, **(e) ***IGFBP4*, and **(f) ***MET *mRNA levels on relapse-free survival among the 33 breast cancer samples.

We then used univariate analysis (log-rank test) to study further the prognostic value of the biomarkers identified. Apart from (as expected) *ESR1*/*ERα *(*P *< 10^-4^), univariate analysis revealed that low expression mRNA levels of *FOS *(*P *< 10^-4^), *BCL2 *(*P *< 10^-4^), *SNCG *(*P *= 0.008), and *IGFBP4 *(*P *= 0.038) were significantly associated with shorter OS (Figure 3 and Table 7). Low expression mRNA levels of *ESR1*/*ERα*, *BCL2*, and *FOS *were also significantly associated with shorter RFS (*P *< 10^-4^; Figure 4 and Table 8), whereas low expression of *SNCG *and *IGFBP4 *was close to statistical significance (*P *= 0.078 and *P *= 0.083, respectively; Figure 4). No significant association between *MET *mRNA level and OS (*P *= 0.879) or RFS (*P *= 0.449) could be identified. In Cox multivariate regression analysis of OS, only the prognostic significance of *ESR1*/*ERα *(*P *= 0.032; hazard ratio [HR] = 0.17, 95% confidence interval [CI] = 0.03 to 0.86) and *FOS *(*P *= 0.049; HR = 0.22, 95% CI = 0.06 to 1.00) persisted (Table 7). Cox multivariate regression analysis of RFS revealed that *BCL2 *(*P *< 10^-4^; HR = 0.10, 95% CI = 0.03 to 0.34) and *FOS *(*P *= 0.038; HR = 0.15, 95% CI = 0.03 to 0.89) were independent prognostic markers and that patients with low mRNA levels of *BCL2 *or *FOS *were at greater risk for relapse (Table 8).

**Table 7:** Univariate and multivariate analysis of the six genes in relation to OS among the 33 breast cancer samples from the independent cohort (Centre Léon Bérard, Lyon, France)

Genes	Univariate (*n* = 33)			Multivariate (*n* = 33)		
		
	HR	95% CI	*P*^a^	HR	95% CI	*P*^a^
*ESR1*	0.09	0.02 to 0.38	<10^-4^	0.17	0.03 to 0.86	0.032
*FOS*	0.12	0.03 to 0.42	<10^-4^	0.22	0.06 to 1.00	0.049
*BCL2*	0.07	0.01 to 0.31	<10^-4^			NS(2)^b^
*SNCG*	0.24	0.08 to 0.75	0.008			NS(3)
*IGFBP4*	0.34	0.12 to 0.98	0.038			NS(1)
*MET*	1.08	0.40 to 2.88	NS	ND^c^	ND	ND

^a^*P *value was considered significant when *P *< 0.05. ^b^NS(i), order in which the variable was deleted from the model. ^c^ND indicates 'not done'; multivariate analysis was done using the variables found to be significant in univariate analysis. CI, confidence interval; HR, hazard ratio; NS, not significant; OS, overall survival.

**Table 8:** Univariate and multivariate analysis of the six genes in relation to RFS among the 33 breast cancer samples from the independent cohort (Centre Léon Bérard, Lyon, France)

Genes	Univariate (*n* = 33)			Multivariate (*n* = 33)		
		
	HR	95% CI	*P*^a^	HR	95% CI	*P*^a^
*ESR1*	0.12	0.04 to 0.36	<10^-4^			NS
*FOS*	0.20	0.08 to 0.51	<10^-4^	0.15	0.03 to 0.89	0.038
*BCL2*	0.08	0.02 to 0.24	<10^-4^	0.10	0.03 to 0.34	<10^-4^
*SNCG*	0.46	0.19 to 1.11	NS	ND^b^	ND	ND
*IGFBP4*	0.46	0.19 to 1.12	NS	ND	ND	ND
*MET*	1.38	0.60 to 3.21	NS	ND	ND	ND

^a^*P *value was considered significant when *P *< 0.05. ^b^ND indicates 'not done'; multivariate analysis was done using the variables found to be significant in univariate analysis. CI, confidence interval; HR, hazard ratio; NS, not significant; RFS, relapse-free survival.

**Figure 5:**
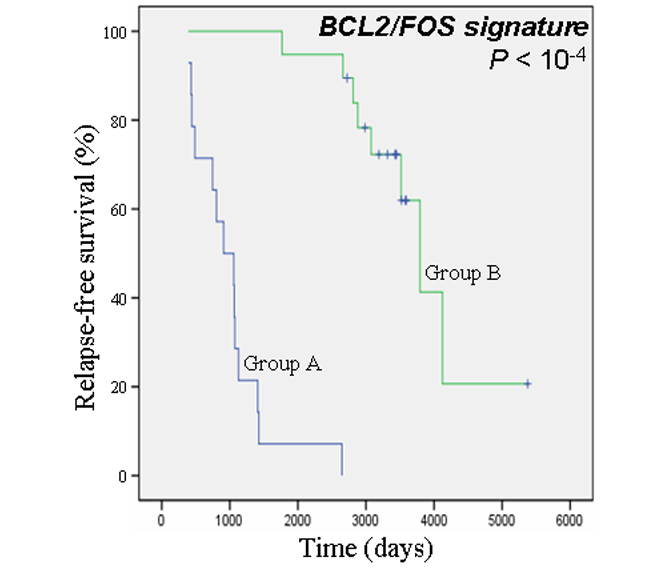
Effect of *BCL2*/*FOS *signature on relapse-free survival. Shown is the effect of the *BCL2*/*FOS *signature on relapse-free survival (Kaplan-Meier curves, log-rank test analysis) among the Centre Léon Bérard cohort. Group A (*n* = 14) contains the breast cancer samples expressing low mRNA levels of both *BCL2 *and *FOS*. Group B (*n* = 19) is composed of the breast cancer samples with high expression levels of at least one of these two genes.

We then defined a two-gene signature based on *BCL2 *and *FOS *mRNA expression levels. This signature was constructed by selecting patients who expressed low mRNA levels of both *BCL2 *and *FOS *(group A; *n* = 14) versus patients exhibiting high expression levels of at least one of these two genes (group B; *n* = 19). Among these 19 patients, concomitant high levels of both *BCL2 *and *FOS *were detected in 15 samples. Concerning the four patients with high expression levels of either *BCL2 *or *FOS*, the Kaplan-Meier curve for RFS could be superimposed over those of the patients having high levels of both *BCL2 *and *FOS*. The resulting Kaplan-Meier curves for RFS according to the *BCL2*/*FOS *signature are illustrated in Figure 5 (univariate analysis, *P *< 10^-4^; HR = 0.014, 95% CI = 0.002 to 0.117).

Finally, we tested the *BCL2*/*FOS *signature with respect to RFS to determine which final model is better. The model with the *BCL2*/*FOS *signature was better fitting (likelihood = 90.35) than the model with *ESR1*/*ERα *alone (likelihood = 109.28; *P *< 10^-4^) or with only *BCL2 *(likelihood = 102.64; *P *= 0.0005) or *FOS *(likelihood = 114.47; *P *< 10^-4^), demonstrating that our two-gene signature has a better prognostic value than *ESR1*/*ERα *alone for RFS. We then tested the model with both *BCL2*/*FOS *and *ESR1*/*ERα *and found that the combination was better fitting (likelihood = 89.82) than the model with *ESR1*/*ERα *alone (likelihood = 109.28; *P *< 10^-4^), demonstrating that the *BCL2*/*FOS *signature could improve the prognostic value of *ESR1*/*ERα *for RFS.

## Discussion

ER immunohistochemistry is a widely available but imperfect test for guiding likely clinical response to tamoxifen treatment. Using clinical material from women with ER-positive breast cancers treated with tamoxifen alone, this study sought biologic markers associated with tamoxifen failure in breast cancer. Transcriptome data identified a specific 47-gene signature associated with tamoxifen failure.

A key issue in gene expression profiling studies is the difficulty in finding overlapped gene expression profiles in independent studies. Compared with the few studies using cDNA array technology to identify molecular markers of tamoxifen resistance, we did not find any overlap between the 47-gene signature of tamoxifen resistance identified and the molecular markers previously identified in the few studies available [13,39-42]. Only one gene (*FLT1*) identified here was present in the 70-gene prognostic signature selected by van't Veer and colleagues [11]. These differences could be due to technical differences in the cDNA array platforms, to the methods of analysis used in each study, or (most probably) to patient inclusion criteria. Fan and colleagues [43] compared the predictions derived from five gene expression profiling studies, and the resulting analysis suggested that even though there was little gene overlap between the different studies, the outcome predictions were similar and probably tracked the same phenotype, illustrated by the same functional clusters.

Among the functional clusters identified in the present study, 34% of the selected genes encode proteins that are involved in immune response, and most of these genes (88%) were under-expressed in the TF group. In accordance with our findings, three other gene expression profiling studies also identified low expression mRNA levels of 'immune response' genes in tamoxifen-resistant tumor samples [13,41,42]. Such downregulations could allow tamoxifen-resistant cells to escape cell death induced by the immune system (for example, cytotoxic T lymphocytes or natural killers cells), or these observations may be a consequence of a modification of the cellular microenvironment in the tamoxifen-resistant tumor samples. However, the dispersal of neoplastic and lymphocyte cells among stromal tissues make this difficult to assess by cDNA array or RTQ-PCR techniques (even with microdissection), but they may be more amenable to the immunohistochemistry approach.

Apart from the 'immune response' cluster, the 'proliferation' cluster is frequently represented in gene expression signatures associated with poor prognosis in breast cancer [15,44,45] or in tamoxifen-treated breast cancer [40,42]. In the present study, 23% of our 47-gene expression signature also belonged to the proliferation cluster and included *IGFBP4*, *MET*, and *SNCG.*

Many investigators have also considered the expression of estrogen-regulated or estrogen-associated genes, because they could provide valuable prognostic or predictive markers of ER-positive breast cancers [32,33,39,45]. In the gene signatures previously identified in tamoxifen-resistant breast carcinoma, 23% to 50% of the genes had been related to estrogens, either as ER targets or ER regulators [13,40,42]. In the present study, 36% of our 47-gene expression signature associated with tamoxifen failure was related to estrogen action, possibly indicating deregulation of the estrogen signaling pathway. Finally, when compared to the chromosomal distribution of all the genes present on the cDNA arrays used in this study, the chromosomal distribution of the 47 selected genes exhibited a significant (*P *< 0.05) over-representation of genes located on chromosomes 6, 11, 17, and 19 (data not shown). Of the 47 genes, 43% were found to be localized to six specific cytobands (6p21, 11q13, 11q23, 17q11-q21, 19p13, and 19q13). Genetic events located on specific cytobands have previously been identified as associated with tamoxifen resistance [13,41,46], and markers for high-level amplification and/or deregulation of expression of genes at 11q13 and 17q12 were strong predictors of reduced survival in breast cancer [47]. Taken together, these observations suggest that deregulation of expression could be associated with genetic alterations that may occur at specific chromosomal loci in the development of tamoxifen resistance.

Based on the results obtained from the cross-analysis of three different statistical analyses performed on the cDNA array data, *ESR1*/*ERα *emerged as the premier gene, in accordance with its well established prognostic and predictive value for endocrine therapy in breast cancer [35-38], and reinforced the gene signature identified in this study. Among the five following top-ranked genes (*MET*, *FOS*, *SNCG*, *IGFBP4*, and *BCL2*), all were validated by RTQ-PCR experiments. However, only two (*IGFBP4 *and *SNCG*) of these five genes were confirmed at the protein level using immunohistochemistry, suggesting first (as observed by others) that the mRNA levels are not always correlated with protein levels, and second that the prognostic/predictive value of a given biomarker can differ with the molecular level investigated (mRNA or protein) [48,49]. In an independent cohort used to assess the pertinence of the biomarkers identified, low mRNAs levels of *ESR1*/*ERα *but also of *FOS*, *BCL2*, *SNCG*, and *IGFBP4 *were significantly associated with tamoxifen failure. The data obtained with the second cohort added new information, indicating that gene expression differences may be inherent to a primary breast tumor before endocrine therapy and might not only reflect deregulation of expression induced by tamoxifen exposure.

Among the six top-ranked genes, this study allowed identification of reduced expression of *FOS *associated with tamoxifen failure. Interestingly, in our cDNA array experiments, two other members of the activator protein-1 complex, namely *JUN *and *JUNB*, were also expressed to lesser degrees in tamoxifen-resistant tumors than in control tumors. *MET *over-expression may be linked to poor clinical outcome in patients with breast cancer [50,51]. High *BCL2 *tumor expression was associated with better outcome in endocrine-treated breast cancers [52-56]. High levels of SNCG (also called *BCSG1 *[breast cancer-specific gene 1]) have been identified in advanced breast carcinomas [57,58] and associated with poor clinical outcome [59]. Recently reported data also demonstrated the role played by SNCG as an ER chaperone and modulator of ER signaling, suggesting that SNCG strongly contributes to the tumorigenesis of ER-positive breast cancer [60], providing evidence for crosstalk between SNCG and ER signaling. IGFBP4 is a member of the IGFBP proteins and appears to be a potent inhibitor of insulin-like growth factor function in several cell lines [61-63]. *IGFBP4*, an estrogen-regulated gene [15,64], is downregulated in tamoxifen-resistant cell lines [65], forms part of a molecular signature of poor prognosis ER-positive breast cancer [32], and could help to identify people who may benefit from endocrine therapy in ovarian cancer [66]. A recent study also revealed that *IGFBP4 *mRNA expression is an independent prognostic factor in breast cancer, and that patients with ER-positive breast cancer with higher levels of *IGFBP4 *tumor mRNA expression and lower levels of *IGFBP5 *mRNA had a better prognosis [67]. However, the number of patients treated with endocrine therapy in that study was too limited to evaluate the predictive value of *IGFBP4 *for endocrine therapy responsiveness.

When assessing the prognostic significance of the markers identified, our analysis revealed several marker dependencies and interactions. For example, although *ESR1*/*ERα*, *FOS*, and *BCL2 *were significant univariate factors for RFS, only *FOS *and *BCL2 *emerged as independent prognostic factors in multivariate analysis. In a previous study, reduced *FOS *gene expression levels were associated with high histologic grade in breast tumors [68]. However, to our knowledge, no study has described any prognostic or predictive value of *FOS *in endocrine-treated patients. A previous immunohistochemistry study suggested that Bcl2 is an independent predictor of breast cancer outcome [69]. In tamoxifen-treated ER-positive patients, low Bcl2 protein expression levels are associated with worse outcome [52-56], and independent prognostic value of Bcl2 protein alone was observed in two studies [54,56]. *BCL2 *and *FOS *are known to be estrogen-regulated genes [15,33,70,71], and we observed in our study a significant positive correlation (Spearman rank correlation test) between *ESR1*/*ERα *mRNA levels and *BCL2 *(*P *< 0.0002) or *FOS *(*P *< 0.01) mRNA levels, both in the first and second cohort of patients (data not shown). Thus, high *BCL2 *levels and/or high *FOS *levels may only reflect aggressiveness of the disease or may be indicative of an intact pathway that is driving tumor growth and that should be sensitive to endocrine therapy. This raises the issue of whether the prognostic value for RFS of the *BCL2*/*FOS *signature is more accurate than *ESR1*/*ERα *mRNA levels. In this study, statistical analysis clearly demonstrated that *BCL2 *alone was more informative than *ESR1*/*ERα *mRNA levels, and that combination of *BCL2 *with *FOS *in a two-gene signature increased the prognostic value of *BCL2*. These data suggest that the biomarkers identified in this study may represent candidate markers that could help in stratifying ER-positive patients, facilitating selection of therapy.

## Conclusions

In this study we identified a gene expression signature and molecular markers associated with tamoxifen failure in breast cancer. RTQ-PCR may provide the best quantitative measure of *IGFBP4*, *BCL2*, *FOS*, *SNCG*, and *MET*, particularly because this technology is in common use in clinical laboratories and could be applied to fine needle aspiration biopsy samples taken sequentially during treatment. Validation at the protein level of SNCG and IGFBP4 using TMAs demonstrated that immunohistochemistry of these proteins may be pursued in the future as a therapeutic decision making tool. Moreover, molecular markers encoding secreted proteins such as IGFBP4, which was validated in this study both by RTQ-PCR and immunohistochemistry, are interesting because they may be investigated in the future in patient serum and offer therapeutic potential. Finally, the low expression levels of *ESR1*/*ERα*, *FOS*, *BCL2*, *SNCG*, and *IGFBP4 *were found to be associated with poor prognosis in an independent cohort of patients exhibiting tamoxifen failure. We also demonstrated the strong prognostic value of the *BCL2*/*FOS *signature in ER-positive patients who relapsed under tamoxifen treatment.

To conclude, the present exploratory study identified new biomarkers of tamoxifen failure that could be helpful in clinical decision making in patients with endocrine-dependant breast cancer. Because the biomarkers identified in this study were confirmed in two independent patient groups, differences between the two cohorts from different geographic location (Ninewells Hospital and Centre Léon Bérard) is unlikely to have influenced the gene signature. However, further work is needed to evaluate the prognostic and/or predictive value of these biomarkers in prospective studies using larger cohorts of patients.

## Abbreviations

CI: confidence interval; ER: estrogen receptor; HR: hazard ratio; IGFBP: insulin-like growth factor binding protein; OS: overall survival; PCR: polymerase chain reaction; RFS: relapse-free survival; RTQ-PCR: real-time quantitative PCR; SNCG: γ-synuclein; TF: tamoxifen failure; TMA: tissue microarray.

## Competing interests

The authors declare that they have no competing interests.

## Authors' contributions

JAV conducted the transcriptome study, performed the RTQ-PCR experiments, and drafted the manuscript. KER, SMH, and CAP conducted the TMA experiments. PR set up and conducted the K nearest neighbor method, and performed the statistical analyses. SEB performed RNA extraction of patient samples and conducted the TMA experiments. AB and SC performed part of the statistical analyses on the validation cohort of tumor samples. CN helped to set up the cDNA array technology. IB helped in the RTQ-PCR investigation. TB selected patients from the Centre Léon Bérard database (Lyon, France). AMT and PAC conceived of the study, its design and coordination, and drafted the manuscript. All authors read and approved the final manuscript.
